# Breast cancer resistance protein identifies clonogenic keratinocytes in human interfollicular epidermis

**DOI:** 10.1186/s13287-015-0032-2

**Published:** 2015-03-24

**Authors:** Dongrui Ma, Alvin Wen Choong Chua, Ennan Yang, Peiyun Teo, Yixin Ting, Colin Song, Ellen Birgitte Lane, Seng Teik Lee

**Affiliations:** Department of Plastic, Reconstructive & Aesthetic Surgery, Singapore General Hospital, Singapore, 168751 Singapore; Skin Bank, Burns Unit, Singapore General Hospital, Singapore, 168751 Singapore; Institute of Medical Biology, A*STAR, Singapore, 138648 Singapore

## Abstract

**Introduction:**

There is a practical need for the identification of robust cell-surface markers that can be used to enrich for living keratinocyte progenitor cells. Breast cancer resistance protein (ABCG2), a member of the ATP binding cassette (ABC) transporter family, is known to be a marker for stem/progenitor cells in many tissues and organs.

**Methods:**

We investigated the expression of ABCG2 protein in normal human epidermis to evaluate its potential as a cell surface marker for identifying and enriching for clonogenic epidermal keratinocytes outside the pilosebaceous tract.

**Results:**

Immunofluorescence and immunoblotting studies of human skin showed that ABCG2 is expressed in a subset of basal layer cells in the epidermis. Flow cytometry analysis showed approximately 2-3% of keratinocytes in non-hair-bearing epidermis expressing ABCG2; this population also expresses p63, β1 and α6 integrins and keratin 14, but not CD34, CD71, C-kit or involucrin. The ABCG2-positive keratinocytes showed significantly higher colony forming efficiency when co-cultured with mouse 3T3 feeder cells, and more extensive long-term proliferation capacity *in vitro*, than did ABCG2-negative keratinocytes. Upon clonal analysis, most of the freshly isolated ABCG2-positive keratinocytes formed holoclones and were capable of generating a stratified differentiating epidermis in organotypic culture models.

**Conclusions:**

These data indicate that in skin, expression of the ABCG2 transporter is a characteristic of interfollicular keratinocyte progentior cells and suggest that ABCG2 may be useful for enriching keratinocyte stem cells in human interfollicular epidermis.

## Introduction

Tissue stem cells or progenitor cells of the epidermis have been identified in several specific niche locations within the pilosebaceous unit of the skin [1-4]. These cells are reported to be slow cycling or rarely cycling cells during homeostasis *in vivo*, with a high capacity for error-free self-renewal, and superior clonogenicity and proliferation capacity [5-8]. Keratinocyte stem cells have been detected by labeling the skin with titrated thymidine or bromodeoxyuridine or by clonal analysis [2,9-11], or *in situ* by immunohistochemistry with antibodies known to label cell populations containing stem cells. Several potential molecular markers for identifying keratinocyte stem cells have been investigated, including β1-integrin, keratin 19, CD34, p63, α6^bri^CD71^dim^, Rac1, MTS24 and survivin [3,12-18]. Although some antibodies to CD71 (transferring receptor) and some integrins have been used to enrich for progenitor containing pools of cells, in most cases it is difficult to use these methods for isolating living cells for stem cell biology studies and clinical use, because cells have to be fixed or permeabilized in order to access the antigens. Moreover there is no clear identification marker for human interfollicular epidermal progenitor cells, although there is a need to identify and characterize these cells for applications in cell and gene therapy [19].

ABCG2, also known as breast cancer resistance protein BCRP1 or CDw338, is a member of the ATP-binding cassette multidrug resistance protein family [20], from the White subfamily. Multidrug resistance proteins are associated with resistance to chemotherapy and are overexpressed in several cancer cell lines. ABCG2 is a transmembrane transporter protein that clears xenobiotics from the cell and so confers drug resistance on cells; it is expressed at high levels in the placenta, where it plays a role in protecting the fetus from xenobiotics. ABCG2 expression is also associated with a side population (SP) cell phenotype observed during fluorescence-activated cell sorting (FACS), due to the ability of ABCG2-expressing cells in many tissues to clear Hoechst 33342 dye from the cells [20-22]. Such ABCG2-expressing SP cells have been demonstrated to show characteristics of stem cells in many tissues and organs, including the hematopoietic system, skeletal muscle, mammary gland and limbus of the eye [23-29], and it has been suggested that expression of the ABCG2 gene is a conserved feature of stem cells from a wide variety of tissues.

ABCG2 expression in the epidermis has not been investigated extensively, although this is a tissue in which there is a high premium on stem cell enrichment (for improved skin autograft generation to treat wounds). A few studies have investigated SP keratinocytes using dye exclusion [30-35]; but it is not known which cell types in human interfollicular epidermis express the ABCG2 transporter protein, and whether such cells possess the characteristics of stem cells [34]. In this study, we investigate the expression of ABCG2 in human epidermis external to hair follicles, and compare the properties of the ABCG2-positive keratinocytes against unsorted keratinocytes in functional assays. We report that within interfollicular and nonhair-bearing epidermis, ABCG2 is specifically expressed in the basal keratinocytes, and ABCG2-positive keratinocytes showed similar stem cell-like properties to other published stem cell marker-identified keratinocyte populations. We demonstrate a proof of concept that ABCG2 is a robust stem cell indicator in human interfollicular keratinocytes that can be practically used to enrich for keratinocyte stem cells.

## Materials and methods

### Isolation and cultivation of keratinocytes from human skin

Normal fresh human skin samples were obtained from surgical waste from plastic surgery operations of healthy subjects, with informed consent from these donors and ethics approval from the ethics committee of Singapore General Hospital. Human skin samples from neonatal foreskins (6 donors) and adult scalp skin (4 donors) were used in this study. Samples were washed in phosphate-buffered saline (PBS) and incubated in 0.25% Dispase II (Roche, Singapore) overnight at 4°C; epidermis was separated from dermis with fine forceps. Epidermis was then minced and incubated in 0.05% trypsin–ethylenediamine tetraacetic acid (Gibco, Invitrogen, Singapore) at 37°C for 15 minutes. Keratinocytes from epidermis were collected and suspended in PBS and filtered on a 40 μm filter (Falcon, Becton Dickinson, Singapore) to obtain a single cell suspension before counting and seeding.

Human skin keratinocytes were seeded at a density of 5 × 10^4^ cells/cm^2^ on a layer of lethally gamma-irradiated 3T3-J2 mouse feeder cells as described previously [36]. The keratinocytes were cultured at 37°C in a 10% carbon dioxide humidified atmosphere. The culture medium used [36] was a mixture of Dulbecco’s modified Eagle’s medium (DMEM; Gibco, Invitrogen) and Ham’s F12 (Gibco, Invitrogen) at a ratio of 3:1, containing 10% fetal bovine serum (FBS; Hyclone, Logan, UT, USA), insulin (5 μg/ml), adenine (0.18 mM), hydrocortisone (0.4 μg/ml), cholera toxin (0.1 nM), triiodothyronine (2 nM), epidermal growth factor (10 ng/ml), l-glutamine (4 mM) and penicillin–streptomycin (50 IU/ml). All reagents were from Sigma (Singapore) unless specified. The culture medium was changed every 3 days, and subconfluent cultures were passaged by treating them with 0.05% trypsin for 8 minutes at 37°C, collecting and counting keratinocytes and replating cells at a density of 5 × 10^3^ cells/cm^2^.

### Immunofluorescence staining of skin

Fresh human skin samples were embedded in OCT embedding media (Sakura, Torrance, CA, USA). Frozen sections of skin (5 μm) were cut with a cryostat (Leica, Houston, TX, USA) and stored at −80°C until use. Immunofluorescence staining was performed as described previously [32]. In brief, human skin sections were fixed with 4% paraformaldehyde at 4°C for 15 minutes; after blocking with 5% goat serum in PBS for 30 minutes, anti-ABCG2 antibody (clone BXP-21; Millipore, Singapore) was used at a 1:25 dilution in PBS at room temperature for 1 hour. After washing with PBS three times, Alexafluor-488 conjugated secondary antibody (1:200; Gibco, Invitrogen) was applied for 1 hour in a dark chamber. After washing with PBS three times, the slides were mounted with Vectashield fluorescent mounting medium containing 4′-6-diamidino-2-henylindole (Vector Labs, Eon Biotech, Singapore) and covered with cover slips. The slides were visualized and photographed with a fluorescent microscope (Axiovert 200; Carl Zeiss, Singapore).

### Immunoblot analysis

Each population of sorted ABCG2-positive, ABCG2-negative keratinocytes and unsorted keratinocytes from human epidermis were collected, centrifuged at 4°C, and the cell pellets were homogenized in RIPA buffer (1% Nonidet P-40, 0.5% sodium deoxycholate, 150 mM sodium chloride, 1 mM phenylmethylsulfonyl fluoride and 1 μg/ml leupeptin) at 4°C. The cell protein lysates was quantified using a BCA kit (Pierce, Rockford, IL, USA). An equal amount of protein was electrophoresed in a 7.5% SDS-polyacrylamide gel, transferred onto a nitrocellulose membrane. The membrane was blocked with 2% bovine serum albumin in Tris-buffered saline for 3 hours, washed with Tris-buffered saline for 1 hour, and incubated with anti-ABCG2 antibody (BXP-21, 1:100; Millipore) diluted in Tris-buffered saline with 0.1% Tween-20 overnight at 4°C. After washing, anti-mouse IgG (horseradish peroxidase-conjugated, 1:2,000; Santa Cruz Technologies, Santa Cruz, Biotechnologies Inc., Zoolem Marketing, Singapore) was applied for 1 hour at room temperature. Finally, chemiluminescence was carried with the Supersignal chemiluminescent substrate (Pierce).

### Flow cytometry analysis and fluorescence-activated cell sorting

Freshly isolated human foreskin epidermis keratinocytes were suspended at a concentration of 10^6^ cells/ml in staining medium (DMEM containing 2% FBS and 10 mM HEPES), incubated with anti-ABCG2 antibody (clone 5D3, 1:25; BD Biosciences, Singapore) for 30 minutes on ice, washed with PBS containing 1% FBS, and then incubated with Alexafluor-488 conjugated rabbit anti-mouse secondary antibody (1:200; Gibco, Invitrogen) for 30 minutes, washed with PBS/1% FBS for 5 minutes three times, and resuspended in staining medium. The cells were kept on ice until flow cytometry analysis was performed. FACS was performed at the National University Medical Institute (Singapore) with a FACSadvantage SE (BD Biosciences, Mountain View, CA, USA) cell sorter.

To characterize the phenotype of ABCG2-positive and ABCG2-negative keratinocytes, the sorted cells were stained for 30 minutes at 4°C with anti-human antibodies against α6-integrin (GoH3), β1-integrin (4B7R), CD34 (8G12), CD71 (C2), keratin 14 (RCK107, phycoerythrin-conjugated, 1:100; all from BD Biosciences, USA), and p63 (4A4, 1:200; Santa Cruz Biotechnology Inc., Zoolem Marketing, Singapore).

### Clonal analysis

After sorting with anti-ABCG2 antibody, single keratinocytes were picked under an inverted microscope as described previously [37], and seeded in a 35 mm Petri dish containing a monolayer of lethally irradiated 3T3 feeder cells. After 7 days of culture, the colonies were identified by phase contrast microscopy. Each colony was then transferred to two 100 mm Petri dishes, with 25% of the cells in one plate (indicator plate for colony-forming efficiency (CFE) assays) and the remaining 75% of the cells in the second plate for further expansion. Selected colonies from the second plate were cultivated serially as above and were passaged as above until they stopped proliferating.

### Colony-forming efficiency assay

To evaluate the CFE, populations of ABCG2-positive, ABCG2-negative and unsorted keratinocytes were collected and 100 cells of each population were plated into 100 mm Petri dishes containing a layer of lethally irradiated 3T3-J2 mouse feeder cells, and cultured as above for 12 days. The culture medium was changed every 4 days. At the end of the culture period, cells were fixed with formalin (see above) and stained with 1% Rhodamine B. The CFE was calculated and expressed as a percentage of the number of plated cells.

### Expansion assay

ABCG2-positive, ABCG2-negative and unsorted keratinocytes were cultured on lethally irradiated 3T3 feeder cells. After keratinocytes reached 70 to 80% confluence, cells were trypsinized and seeded at a density of 5,000 cells/cm^2^. Cultures were serially passaged until the proliferation capacity of the keratinocytes was exhausted. The number of population doublings was calculated according to the following formula:

Population doublings = (log *N* / *N*_0_) / log_2_

where *N* represents the total number of cells obtained at each passage and *N*_0_ represents the number of cells plated at the start of the experiment.

### RNA isolation and RT-PCR analysis

Total RNA from ABCG2-positive, ABCG2-negative and unsorted keratinocytes was isolated using the RNeasy kit (Gibco, Invitrogen). The RNA was quantified by its absorption at 260 nm using a NanoDrop spectrophotometer (ND-1000; NanoDrop Technologies, Thermo Scientific, Singapore) and stored at −80°C until use. A housekeeping gene, glyceraldehyde-3-phosphate dehydrogenase, was used as an internal control. The mRNA expression level of different molecular markers was analyzed by semiquantitative RT-PCR as described previously [20]. Briefly, 1 μg total RNA was used to synthesize first-strand cDNAs with Superscript first-strand System (Gibco, Invitrogen). PCR amplification was performed with specific primer pairs designed from published human gene sequences for different markers [20]. The fidelity of the RT-PCR products was verified by comparing their size with expected cDNA bands and sequencing the PCR products. The PCR products were also visualized on 1.8% agarose gel.

### Reconstructing epidermis with organotypic culture

Glycerol-preserved allogenic skin (Euro Skin Bank, EA Beverwijk, the Netherlands) was used and washed in PBS containing penicillin–streptomycin (50 IU/ml). The epidermis was removed mechanically after three cycles of snap-freezing and thawing. The de-epidermized dermis was cut into 2 × 2 cm squares, and a 1 cm diameter stainless steel ring was placed on the reticular side of each dermis square. Normal primary human dermal fibroblasts were seeded onto the dermis at 5 × 10^5^ cells per ring and grown for 1 day in DMEM supplemented with 10% FBS, l-glutamine (4 mM) and penicillin–streptomycin (50 IU/ml). ABCG2-positive and ABCG2-negative keratinocytes were then plated on top at 2 × 10^5^ cells per ring respectively, and cultured in keratinocyte culture medium for 4 days to reach a confluent monolayer. The cultures were then raised to the air–liquid interface and cultured for a further 10 days. The cultures were embedded in OCT and snap frozen in liquid nitrogen; frozen sections were cut at 5 μm and stained with hematoxylin and eosin.

### Preparation of cultured epidermal grafts and grafting to nude mice

The protocol for preparation of cultured epidermal grafts was adopted from a previous study [38]. Cultured epidermal grafts were prepared in a six-well plate. In brief, 10 ml pig plasma was mixed with 100,000 human skin dermal fibroblasts (0.5 million/ml) supplemented with 13 ml of 0.9% NaCl solution. This mixture (total 23 ml) was carefully added to 2 ml of 1% CaCl_2_ solution, and the mixture solution was pipetted into a six-well plate at 4 ml/well. The six-well plate was transferred to a carbon dioxide incubator at 37°C, where the mixture solidified within 15 minutes. Then 3 ml DMEM + 10% FBS was added to each well. ABCG2-positive, ABCG2-negative keratinocytes, unsorted keratinocytes or α6-integrin-positive keratinocytes were then seeded onto the plasma gel 16 to 24 hours later and cultured to confluence.

All animal studies were carried out with the approval of the Singhealth Institutional Animal Care and Use Committee in Singhealth Experimental Medical Center under specific pathogen-free conditions. Nude athymic BALB/c nu/nu mice 6 to 8 weeks old (purchased from Animal Resources Center, Perth, Western Australia) were used as skin graft recipients. Each animal was aseptically cleansed and a full-thickness 3 cm circular skin wound was made on the dorsum, into which a cultured epidermal graft, detached from the culture well, was placed. The wound was covered with the excised skin that had been de-vitalized by four cycles of freezing and thawing, sutured in place to act as wound dressing to protect and fix the skin grafts. To further protect the graft site, surgical normal gauze was applied on top of the devitalized skin, followed by several layers of standard dressing gauze. Antibiotics (Baytril®, enrofloxacin: Bayer Healthcare, Singapore; 120 mg/kg in drinking water) were given at the time of grafting and were continued for the next 5 days. After transplantation, animals were observed each day, and the wound dressings were changed every 2 days. The wound dressings were removed when the regenerated epidermis was robust enough, usually 3 to 4 weeks after transplantation. The mice were further maintained up to 20 weeks. The regenerated human epidermal grafts were biopsied at different time intervals, snap frozen in liquid nitrogen, and 8 μm frozen sections were prepared for hematoxylin and eosin staining.

### Statistical analysis

The CFE data were calculated as a mean value (triplicate) ± standard error of the mean, as indicated. Differences between mean values were analyzed with Student’s *t* test, and *P* <0.05 was considered statistically significant.

## Results

### ABCG2 expression is restricted to the basal layer of human skin epidermis

**Figure 1:**
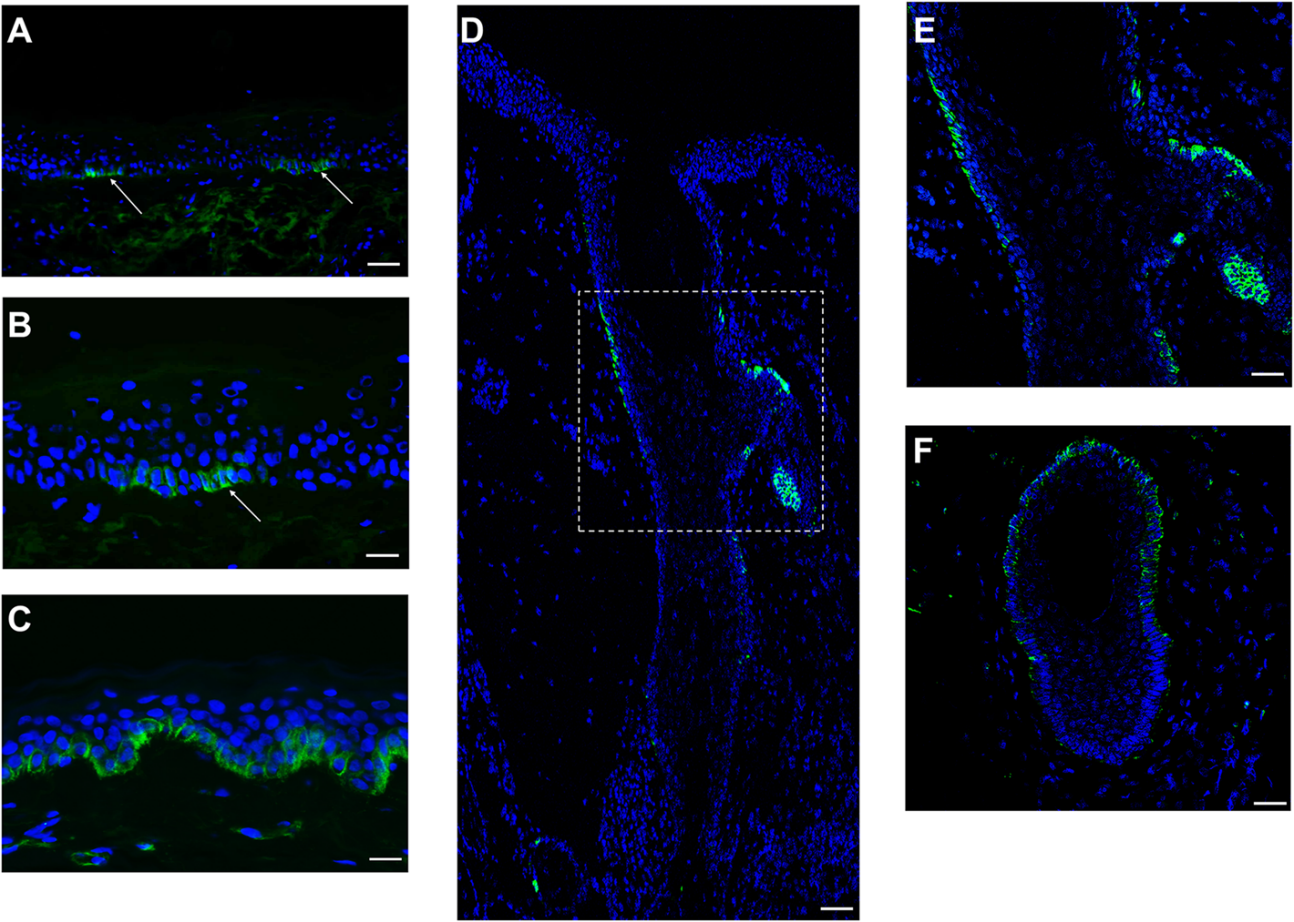
**Localization of ABCG2 in human interfollicular skin and hair follicle by immunofluorescence staining of human interfollicular epidermis and hair follicle with anti-ABCG2 antibody (clone BXP-21).** Bright green cell membrane staining of ABCG2 was seen in clusters of small cells of basal layer keratinocytes (white arrow) in human skin **(A, B)** and hair follicle **(D, E, F)**, whereas suprabasal keratinocytes were unstained stained. (B) High magnification of (A). (E) High magnification of (D). **(C)** Human interfollicular skin was stained with anti-keratin15 antibody (clone LHK15), and most basal cells were positively stained (green) on their cell membrane. Cell nuclei were counterstained with 4′-6-diamidino-2-phenylindole DAPI: (blue). Original magnification: A, D, F, ×10; and B, C, E, ×20. Scale Bars: A, D, F, 50 μm; and B, C, E, 50 μm.

**Figure 2:**
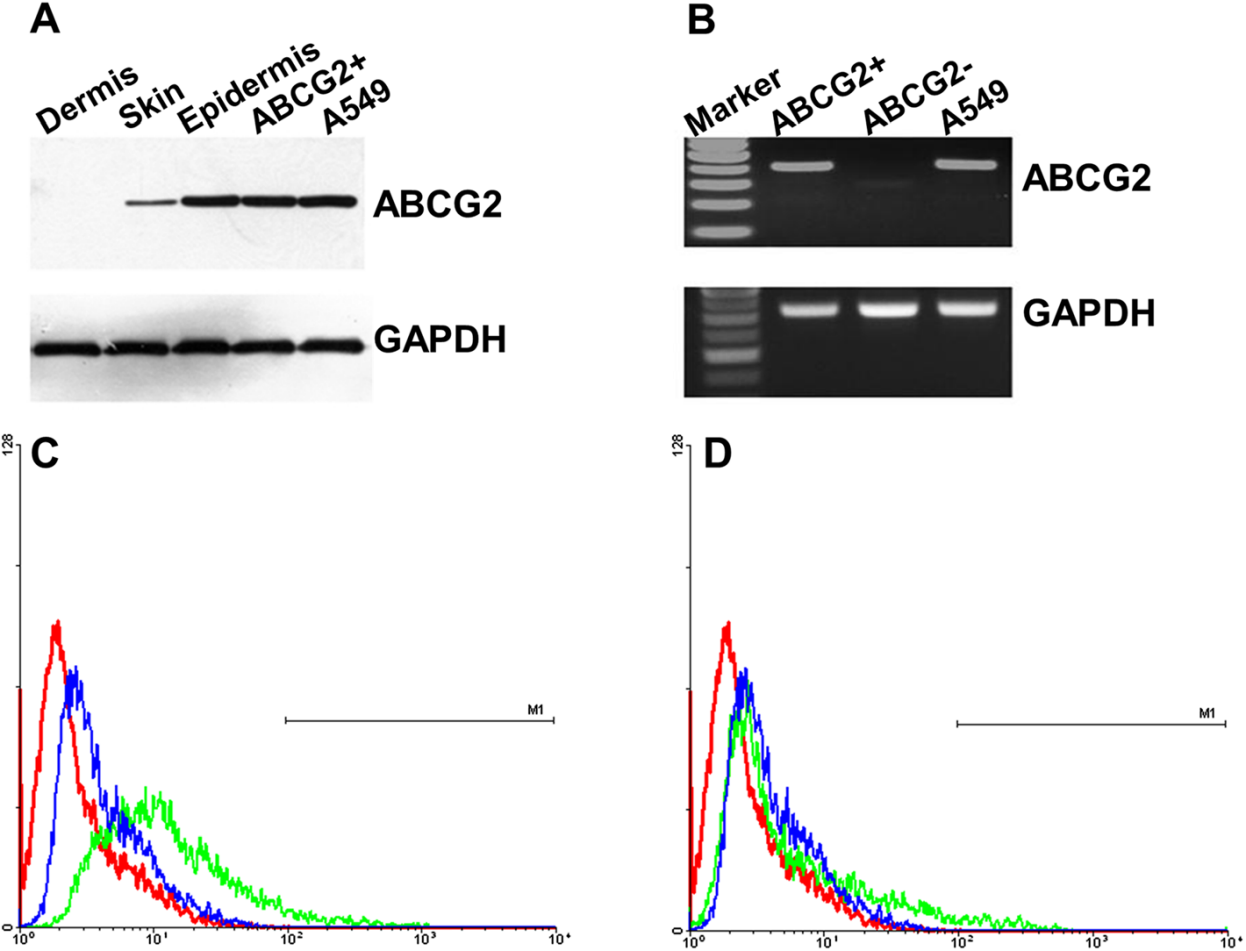
**Localization of ABCG2 in human skin by western blot, RT-PCR assay and flow cytometry analysis. (A)** Western blot analysis of ABCG2 expression in human skin. Epidermis and positive control (A549 cell line) expressed ABCG2, dermis tissue did not; a lower level of ABCG2 staining was detected in whole unfractionated skin. **(B)** RT-PCR analysis of ABCG2 mRNA expression in sorted keratinocytes. Higher expression of ABCG2 mRNA (379 base pairs (bp)) was shown in ABCG2-positive keratinocytes sorted by fluorescence-activated cell sorting, while lower expression of ABCG2 mRNA was shown in ABCG2-negative keratinoctyes. A 100 bp DNA ladder is shown in the first left lane. Glyceraldehyde-3-phosphate dehydrogenase (GAPDH; 498 bp) was used as an internal control. **(C)** Fresh isolated epidermal keratinocytes were incubated with anti-ABCG2 antibody (clone 5D3), and Alexafluor-488 conjugated secondary antibody was used. ABCG2-positive cells (green line, region M1) accounted for approximately 2.7%; isotype control, blue line; blank control, red line. **(D)** Primary cultured epidermal keratinocytes were harvested and incubated with anti-ABCG2 antibody; approximately 2.8% of cells showed ABCG2-positive staining (green line, region M1). The flow analyses were repeated five times, and the results were averaged.

By immunohistochemistry of human skin sections, ABCG2 protein was detected in a range of sites previously associated with locations rich in keratinocyte progenitor/stem cells in the hair follicle, as well as in the small cells of the basal layer keratinocytes in interfollicular epidermis (Figure 1A,F), and both membrane-associated and cytoplasmic staining were observed. No ABCG2 expression was detected in suprabasal keratinocytes (Figure 1B). There was minimal ABCG2 staining in the dermis. The staining pattern is similar to that of several other progenitor markers in the epidermis, such as the transcription factor p63: positive staining was seen in isolated small clusters of basal keratinocytes, interspersed with large areas of unstained cells [15]. This staining pattern is similar to previous reports of ABCG2 staining in mouse skin and human limbus [28,32]. Immunoblot analysis revealed a single protein band with a molecular weight of approximately 72 kDa that was detected in any samples from whole skin, epidermis, ABCG2-positive keratinocytes or A549 cells (Figure 2A). As expected, the highest expression of ABCG2 was detected in ABCG2-positive keratinocytes sorted by FACS, followed by epidermis and whole skin, while dermis expressed little if any ABCG2. These results show that in human interfollicular epidermis, ABCG2 is predominantly expressed in the basal layer keratinocytes.

To estimate the proportion of ABCG2-expressing basal keratinocytes in skin epidermis, flow cytometry analysis was performed using the anti-ABCG2 antibody (clone 5D3; eBioscience, BD Biosciences, Singapore). ABCG2-positive keratinocytes accounted for 2.7 ± 0.6% (mean ± standard deviation, *n* = 5; Figure 2C, region M1) of freshly isolated keratinocytes, and 2.8 ± 0.3% (mean ± standard deviation, *n* = 5; Figure 2D, region M1) of primary epidermal keratinocyte cultures. ABCG2 expression in the cells sorted by FACS was confirmed by comparing the levels of ABCG2 mRNA detected by RT-PCR in ABCG2 antibody-positive and antibody-negative populations. ABCG2 mRNA was highly expressed in the antibody-positive population and A549 cell line, which was taken as a positive control, whereas mRNA was barely detectable in the ABCG2 protein-negative population. There was no difference in glyceraldehyde-3-phosphate dehydrogenase expression among these three groups (Figure 2B).

### Phenotype of ABCG2-positive keratinocytes

**Figure 3:**
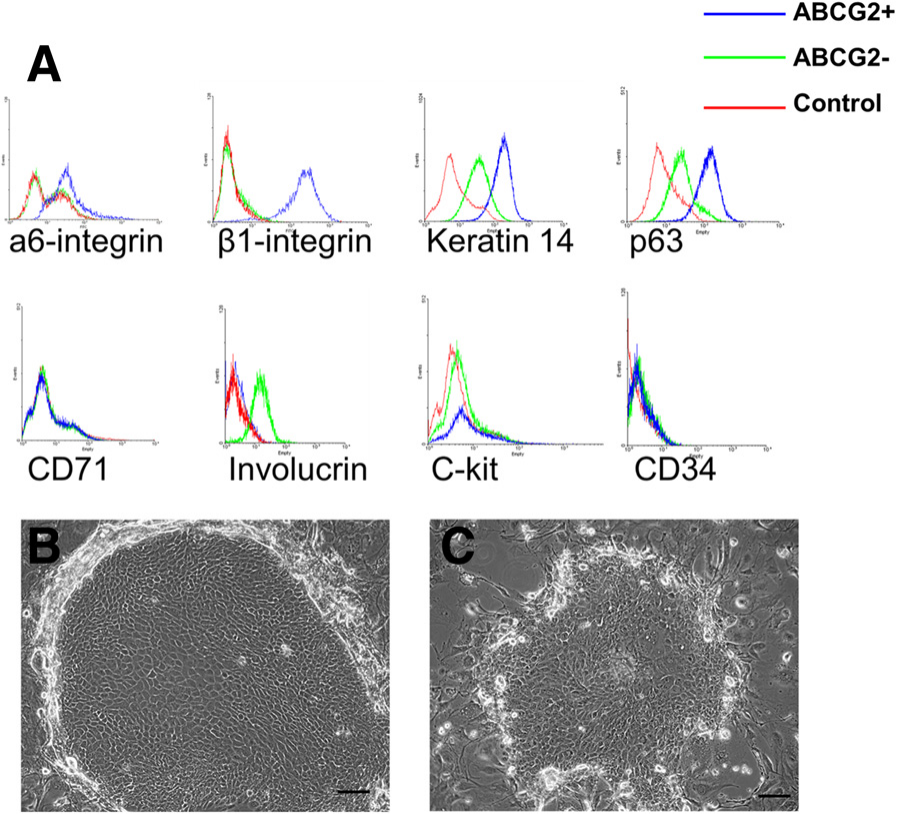
**Phenotype of ABCG2-positive keratinocytes. (A)** Expression of stem cell markers in human epidermal keratinocytes. ABCG2-positive and ABCG2-negative keratinocytes were sorted separately as in Figure 2. ABCG2-positive cells (blue line) were more strongly stained with antibodies to α6-integrin, β1-integrin, keratin 14 and p63 than ABCG2-negative cells (green line); CD71 and involucrin staining was weak in ABCG2-positive cells; both ABCG2-positive and ABCG2-negative cells were unstained for CD34 and C-kit. Isotype control, red line. **(B, C)** Colonies formed by ABCG2-positive keratinocytes and ABCG2-negative keratinocytes. ABCG2-positive and ABCG2-negative keratinocytes were sorted and seeded onto a lethally irradiated 3T3 feeder layer, and allowed to grow for 7 days. (B) ABCG2-positive keratinocytes formed a large colony, composed of small and actively growing keratinocytes. (C) ABCG2-negative keratinocytes formed small colonies, with irregular margin and big keratinocytes, indicating an abortive colony. Scale bars: B, C, 100 μm.

The phenotype of ABCG2-positive and ABCG2-negative keratinocytes was determined by examining the expression of several known growth and differentiation status markers. As shown in Figure 3A and Table 1, ABCG2-positive keratinocyte populations were also strongly stained with antibodies to α6-integrin, β1-integrin, keratin 14 and p63, compared with ABCG2-negative keratinocytes, whereas ABCG2-positive keratinocytes were weakly stained by CD34, CD71, C-Kit and involucrin antibodies when compared with ABCG2-negative keratinocytes. These data suggest that ABCG2-positive keratinocytes express high levels of several potential stem cell markers, while expressing low levels of differentiation markers.

**Table 1:** **Mean fluorescence intensity of three groups of keratinocyte stained with molecular markers**

**Marker**	**ABCG2-positive**	**ABCG2-negative**	**Control**
α6-integrin	89 ± 5.6	6.8 ± 0.6	7.2 ± 0.8
β1-integrin	663 ± 58.4	5.8 ± 0.7	6.3 ± 0.6
Keratin 14	557 ± 68.1	84 ± 70.4	9.5 ± 8.3
p63	398 ± 52.4	65 ± 7.8	8.8 ± 7.6
CD71	6.5 ± 0.7	6.9 ± 0.8	5.8 ± 0.4
Involucrin	5.3 ± 0.4	66 ± 5.7	4.9 ± 0.5
C-kit	8.4 ± 0.5	7.8 ± 0.6	6.3 ± 0.4
CD34	3.4 ± 0.2	4.1 ± 0.3	1.9 ± 0.3

Data presented as mean ± standard error of three biological replicates from ABCG2-positive, ABCG2-negative and control group keratinocytes.

### Colony-forming efficiency assay and clonal analysis

Next we examined the CFE and the colony morphologies produced by each population of ABCG2-positive/negative keratinocytes sorted by FACS. ABCG2-positive and ABCG2-negative keratinocytes were seeded at a density of 100 cells per 100 mm culture containing a layer of lethally irradiated 3T3 feeder cells, and keratinocytes were cultured for 12 days. The colony types produced by the two sorted keratinocyte populations were very obviously different. Figure 3B,C shows examples of the different colonies observed in ABCG2-positive and ABCG2-negative keratinocyte cultures. Large colonies were observed in ABCG2-positive cultures but not in the ABCG2-negative cultures (Figure 3B). Also, most of the colonies formed by the ABCG2-positive population were composed of small actively growing keratinocytes (Figure 3B), while most of the cells in ABCG2-negative colonies were very large and mostly abortive within 4 weeks (Figure 3C).

**Figure 4:**
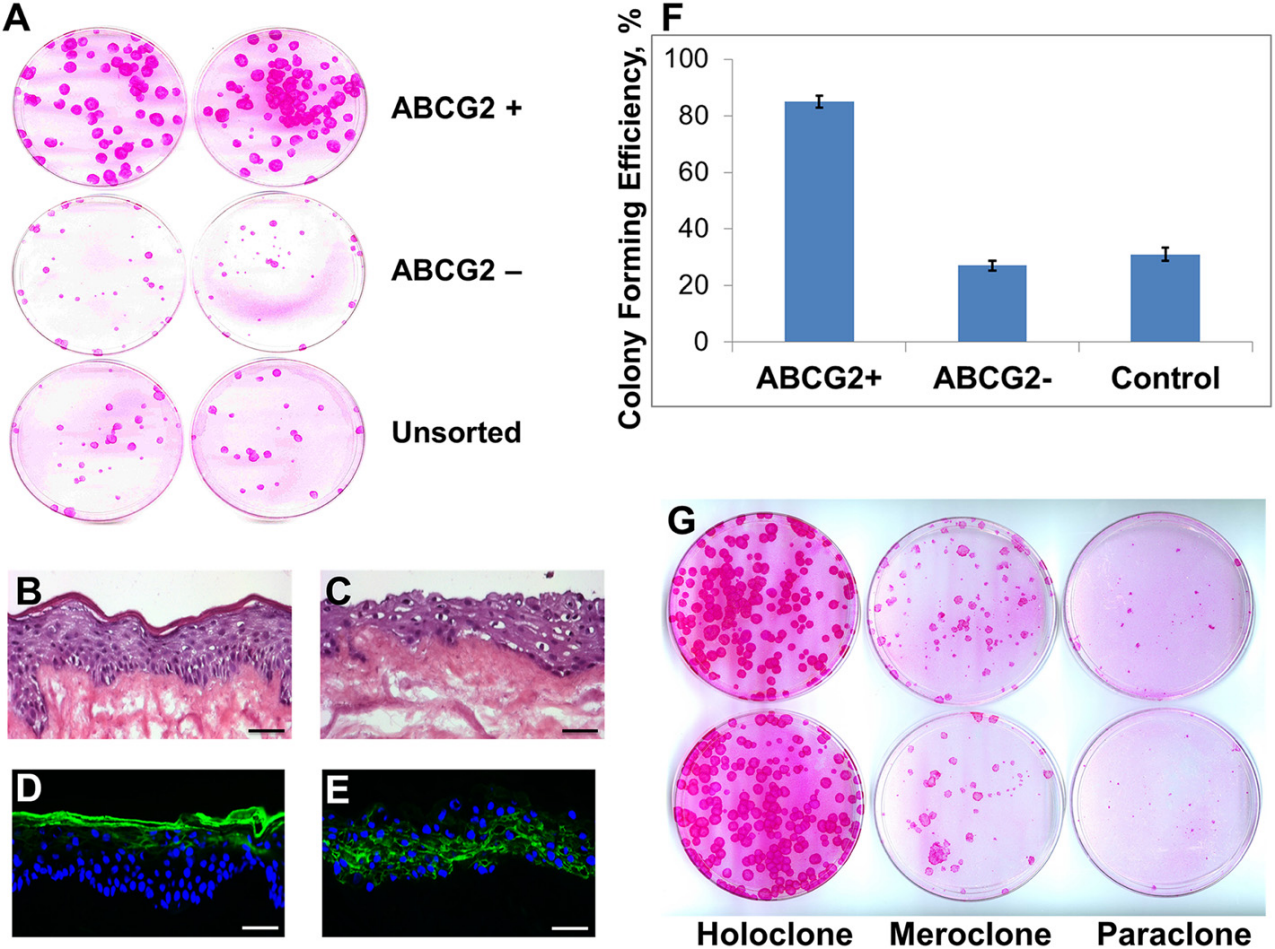
**Colony-forming efficiency assay and skin organotypic assay. (A), (F)** Colony-forming efficiency (CFE) of ABCG2-positive, ABCG2-negative and unsorted keratinocytes. (A) ABCG2-positive and ABCG2-negative keratinocytes were sorted as in Figure 3, 100 cells of each population were seeded in a 100 mm Petri dish preseeded with a lethally irradiated 3T3 feeder layer, and CFE was evaluated at day 12. (F) The ABCG2-positive cells showed a greater number of colonies than the ABCG2-negative cells (CFE, 85% ± 2.1% vs. 27% ± 1.8%, *P* <0.01, *n* = 5), whereas there was no difference between ABCG2-negative cells and unsorted cells (CFE, 27% ± 1.8% vs. 31 ± 2.3%, *P* >0.05, *n* = 5). **(B) to (E)** Epidermal reconstruction (organotypic) assay of ABCG2-positive and ABCG2-negative keratinocytes. Human epidermal ABCG2-positive and ABCG2-negative keratinocytes were sorted as in Figure 3 and plated onto a de-epidermized dermis (DED) substrate. (B) ABCG2-positive cells were seeded on top of DED; 1 week after seeding, stratified epidermis was formed, with a typical stratum granulosum and stratum corneum. (C) ABCG2-negative cells were seeded on top of DED; 1 week later, a disordered multilayered tissue had formed. Scale bars: 100 μm. (D) Immunostaining of involucrin in ABCG2-positive culture showed strong positive staining (green) in the suprabasal layer. (E) Weak and abnormal staining for involucrin was detected in ABCG2-negative culture. **(G)** Clonal analysis of ABCG2-positive epidermis keratinocytes. ABCG2-positive cells were sorted as in Figure 3, and a total of 300 single cells from five donors were picked and inoculated into a 35 mm Petri dish preseeded with lethally irradiated 3 T3 cells. Seven days later, one-quarter of each resulting colony was trypsinized, and transferred to an indicator dish. These dishes were fixed, stained and analyzed 12 days later. The holoclones, meroclones and paraclones shown here are typical figures. Scale bars: D, E, 50 μm.

As shown in Figure 4A,F, the CFE values for ABCG2-positive, ABCG2-negative and unsorted keratinocytes were 85% ± 2.1%, 27% ± 1.8% and 31 ± 2.3% respectively. The ABCG2-positive keratinocytes exhibited statistically higher CFE and larger colony sizes, as compared with ABCG2-negative keratinocytes and unsorted cells (*P* <0.01, *n* = 5; Figure 4F). No difference in CFE was found between ABCG2-negative cells and unsorted cells (*P* >0.05, *n* = 5; Figure 4F).

**Figure 5:**
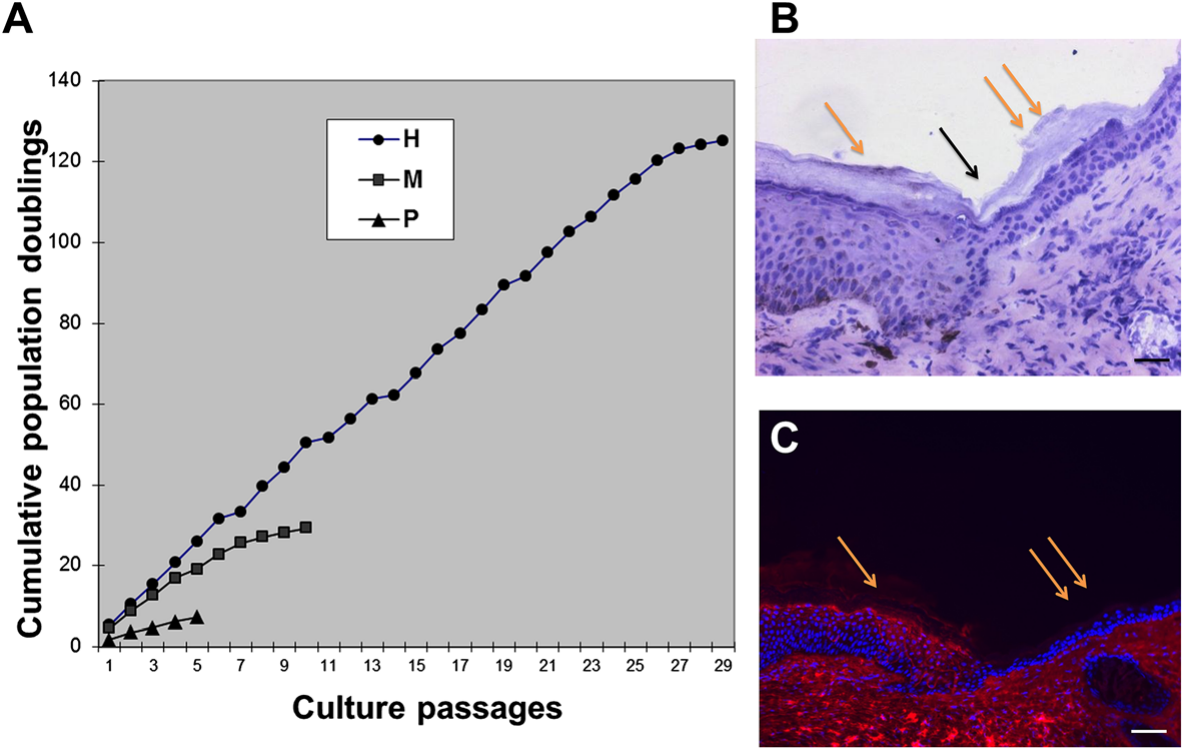
**Proliferation potential of primary keratinocytes in long-term culture and immunostaining of transplanted skin grafts. (A)** ABCG2-positive keratinocytes were sorted; holoclones, meroclones and paraclones were identified, and subcultured serially on a lethally irradiated 3T3 feeder layer until their growth potential was exhausted. Triplicate cultures in each keratinocyte clones were cultured, and a total of five keratinocyte clones were analyzed. The number of cumulative population doublings was calculated using the following formula: population doublings = (log *N* / *N*_0_) / log_2_, where *N* represents the total number of cells obtained at each passage and *N*_0_ represents the number of plating cells at the beginning. H, holoclone; M, meroclone; P, paraclone. The image is a typical graph. **(B), (C)** Immunostaining of anti-involucrin monoclonal antibody was used to detect transplanted human grafts in mouse skin samples. Positive staining (red) was observed in human grafts (single orange arrow, C), while negative staining was observed in mouse skin (double orange arrow, C), and distinct separation was observed (black arrow, B). Scale bars: B, C, 50 μm.

To investigate the clonogenic potential of ABCG2-positive keratinocytes versus ABCG2-negative keratinocytes, single cell clones from each population were isolated, expanded and assayed for their ability to generate clones with high proliferation potential as would be expected for a stem cell. In total, 300 clones from five donors were analyzed for their ability to generate colonies identified as holoclones, paraclones and meroclones [37]. We found that 73.7 ± 1.2% of clones from ABCG2-positive keratinocytes were holoclones, whilst meroclones and paraclones represented less than 27%. For ABCG2-negative cells, the holoclones were less than 4%, and 95% of the clones were meroclones and paraclones (Table 2, Figure 4G). The holoclones, meroclones and paraclones [34] obtained from the same donors were then cultivated serially to evaluate their proliferative capacity (Table 3). The holoclones were found to produce 120 to 140 cell generations before they stopped proliferating, while meroclones were able to undergo 20 to 42 cell divisions and paraclones only underwent five to 10 cell divisions before exhausting their proliferation capacity (Figure 5A).

**Table 2:** **Clonogenic potential of ABCG2-positive versus ABCG2-negative human interfollicular primary keratinocytes**

**Keratinocyte clone**	**ABCG2-positive cells**			**ABCG2-negative cells**		
	**H**	**M**	**P**	**H**	**M**	**P**
NK1213	75	20	5	3.33	35	61.67
NK0428	71.67	25	3.33	5	31.67	63.33
NK0609	73.33	18.33	8.34	1.67	26.67	71.67
NK0411	78.33	16.67	5	1.67	30	68.33
NK0914	70	23.33	6.67	3.33	25	71.67
Mean	73.67 ± 1.21	20.67 ± 1.55	5.67 ± 0.85	3 ± 0.62	29.67 ± 1.78	67.33 ± 2.08

Data presented as percentage or mean ± standard error. H, holoclone; M, meroclone; P, paraclone.

**Table 3:** **Proliferation capacity of primary ABCG2-positive keratinocyte clones in long-term culture**

**Keratinocyte clone**	**Number of samples**	**Age of donors (years)**	**Culture days**	**ABCG2-positive cells PD**
NK1213	3	23	140 ± 8	124.3 ± 1.8
NK0428	4	35	115 ± 12	136.2 ± 3.4
NK0609	3	45	130 ± 10	129.5 ± 2.2
NK0411	3	28	125 ± 12	131.8 ± 2.1
NK0914	4	46	135 ± 10	136.4 ± 2.7

Data presented as mean ± standard error. PD, population doublings.

### Epidermal reconstruction assay

We also compared the ability of each population to differentiate and regenerate a multilayered epidermis. ABCG2-positive and ABCG2-negative keratinocytes sorted by FACS were plated onto a de-epidermized dermis substrate. As shown in Figure 4B, the ABCG2-positive cells were able to form a stratified epithelium typical of a normal epidermal structure and cellular differentiation: a basal layer with polygonal keratinocytes oriented perpendicularly to the underlying de-epidermized dermis, three to four layers of spinous cells, three to four layers of granular cells characterized by the presence of keratohyalin granules, and layers of anucleate, flattened cornified cells forming a compact stratum corneum. In contrast, ABCG2-negative cells only formed an irregular and disorganized epidermis with two to three layers of keratinocytes (Figure 4C). The organotypic structure formed by the ABCG2-positive cells was thicker than a normal steady-state *in vivo* epidermis, and this was attributed to the high proportion of clonogenic cells used to initiate the organotypic construct. Immunostaining of the culture from these two groups with anti-involucrin antibody showed strong positive staining (green) in suprabasal layers in ABCG2-positive samples (Figure 4D), while strong staining of involucrin was also detected in ABCG2-negative groups (Figure 4E), but it is of early onset, suggesting disorganization of differentiation and loss of terminal differentiation. This result indicates that the capacity for differentiation, as well as the clonogenic capacity, is retained in ABCG2-positive keratinocytes, and thus these two cell populations can be clearly distinguished on the basis of this functional test.

### Transplantation of cultured epidermal grafts onto nude mice

**Figure 6:**
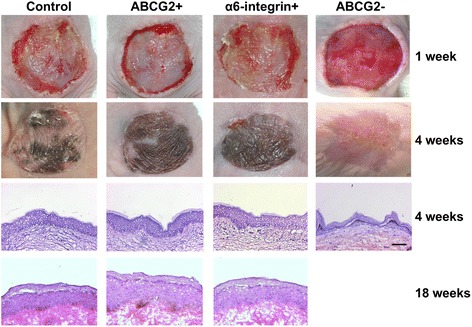
**Transplantation of cultured epidermal grafts onto nude mice.** Human ABCG2-positive and ABCG2-negative keratinocytes were sorted out and used to prepare cultured epidermal grafts; unsorted keratinocytes and α6-integrin-positive keratinocytes were also used for comparison. All grafts survived for the duration of transplantation, and no signs of necrosis were observed. All grafts from four groups of animals showed no blistering, and the take rate was excellent. However, the whitish-covered grafts in ABCG2-negative groups were observed to be much thinner than in the other three groups. Four weeks after grafting, persistent human epidermis was observed in all groups except in the ABCG2-negative group in which the mouse epidermis was observed to have populated the skin wound. Eighteen weeks after grafting, well organized human epidermis, with all histological cell layers represented, was observed in ABCG2-positive, a6-integrin-positive and control groups, which indicated the complete maturation and differentiation of epidermal cells. Melanocytes were also observed within the basal layer, and distinct rete ridge structures could also be seen. Scale bars: 50 μm.

**Figure 7:**
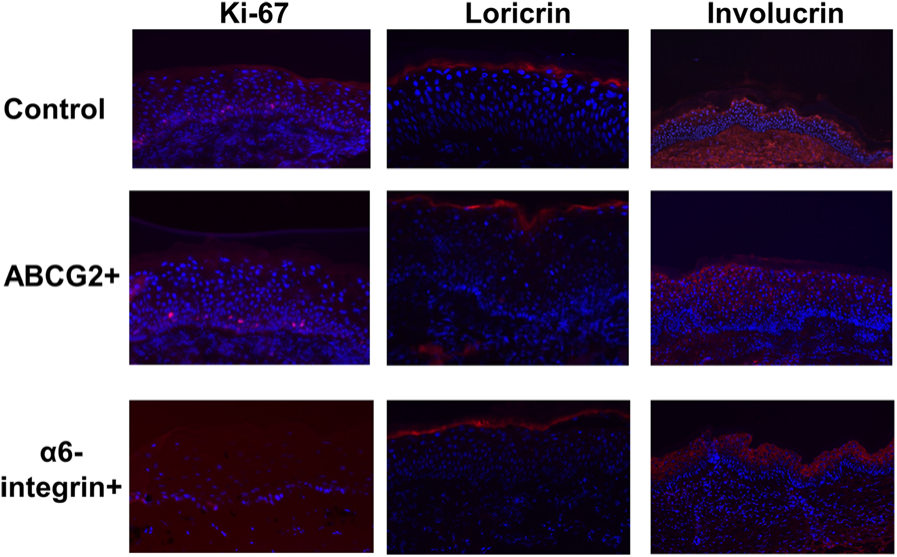
**Immunostaining of transplanted skin grafts at 18 weeks after transplantation.** Staining of differentiation markers, involucrin and loricrin, showed signs of mature and well differentiated human epidermis (Figure 5). Interestingly, for the staining of the cell proliferating marker Ki-67, the ABCG2-positive group showed a cluster of positive staining in basal keratinocytes, while relatively sparse positive staining was observed in the control and α6-integrin-positive groups. Scale bars: 50 μm.

To determine the long-term epidermal regeneration capacity of ABCG2-positive keratinocytes, ABCG2-positive and ABCG2-negative keratinocytes were sorted and used to prepared cultured epidermal grafts; unsorted keratinocytes and α6-integrin-positive keratinocytes were also used for comparison. These grafts were transplanted onto dorsum full-thickness wounds in nude mice, and the take of grafts was assessed and followed by histological analysis (Figures 6 and 7). Immunostaining of anti-involucrin monoclonal antibody was used to detect human grafts in mouse skin samples (Figure 5B,C), and positive staining (green) was observed in human grafts (single orange arrow, Figure 5C) while no staining was observed in mouse skin (double orange arrow, Figure 5B,C), and distinct separation was observed (black arrow, Figure 5B).

All grafts survived for the duration of transplantation, and no signs of necrosis were observed (Figure 6). The gross appearance of grafts changed from shiny (before grafting) to whitish colored, which indicated the formation of stratum corneum on each graft, induced by contact with air. All grafts from four groups of animals showed no blistering, and the take rate was excellent. However, the whitish-covered grafts in the ABCG2-negative group were observed to be much thinner than in the other three groups. Four weeks after grafting, persistent human epidermis was observed in three of the four groups (grafts derived from ABCG2+, a6-integrin+, or unselected (control) cells), whereas in the ABCG2-negative group the mouse epidermis was observed to have populated the skin wound with thickened stratum corneum and thinner epidermis (Figure 6). Eighteen weeks after grafting, well organized human epidermis, with all histological cell layers represented, was observed in the ABCG2-positive, a6-integrin-positive and control groups, which indicated the complete maturation and differentiation of epidermal cells. In the ABCG2-positive group, the epidermis consisted of 14 to 16 cell layers, which is significantly thicker than those in the control and a6-integrin groups (eight to 10 cell layers). Melanocytes were also observed within the basal layer, and rete ridge structures could also be seen. The staining of epidermal differentiation markers, including involucrin and loricrin, performed at 18 weeks after transplantation showed signs of mature and well differentiated human epidermis (Figure 5). Interestingly, for the staining of the cell proliferating marker Ki-67, the ABCG2-positive group showed a cluster of positive staining in basal keratinocytes, while relatively sparse positive staining was observed in the control and α6-integrin-positive groups. This observation suggests that there were more proliferative keratinocytes within the basal layer of grafts derived from cells selected as ABCG2-positive, compared with grafts derived from the control (unfractionated keratinocytes) or the α6-integrin-positive selected cells.

## Discussion

This is the first report on the use of ABCG2 to isolate progenitor-enriched cells from human interfollicular epidermis. We show that, outside of the hair follicles, ABCG2 transporter expression is restricted to the basal layer of human epidermis, and is not expressed in the suprabasal layer. The ABCG2-positive keratinocytes account for 2.1 to 3.3% of the total basal epidermal keratinocytes. These cells are also p63-positive, β1 integrin-positive, α6 integrin-positive, keratin14-positive, CD34-negative, CD71-negative, C-kit-negative and involucrin-negative. The ABCG2-positive keratinocytes showed significantly higher CFE on 3T3 feeder layers than ABCG2-negative keratinocytes. The ABCG2-positive keratinocytes also showed extensive proliferative capacity in long-term culture, and most of the freshly isolated ABCG2-positive keratinocytes formed holoclones. They can also form pluristratified epidermis in an organotypic culture model. Our data indicate that ABCG2 is exclusively expressed by a subset of basal keratinocytes in human interfollicular epidermis, and that this population of cells possesses properties of keratinocyte stem cells, suggesting that ABCG2 might be a useful marker for enriching for keratinocyte stem cells in human interfollicular epidermis.

Previous studies have shown that ABCG2 expression is present in both cytoplasm and cell membrane, and is only found in stem cells [20,22,28,39,40]. Our immunofluorescence staining and western blot analysis showed that ABCG2 expression was specifically localized to certain basal keratinocytes in human interfollicular epidermis, a small subpopulation of the basal layer keratinocytes, which is consistent with previous reports of ABCG2 staining in mouse skin [32,39]. We found that the proportion of ABCG2-expressing cells in interfollicular epidermis keratinocytes was higher than in other tissues examined, such as bone marrow, which was reported to be 0.005 to 0.1% [21,23]. *In vivo* studies in mice suggest that keratinocyte progenitor cells make up around 4% of the basal layer keratinocytes [13]. Our data of 2.1 to 3.3% ABCG2-positive keratinocytes is consistent with these previous observations.

Many molecular markers for epithelial stem cells have been investigated, including the transcription factor p63 [15], β1-integrin [3,4], α6-integrin and transferrin receptor CD71 [13], CD34 [31,41], keratin 19 [12] and ABCG2 [28]. We found that p63, β1-integrin, α6-integrin and keratin 14 were highly expressed in ABCG2-positive keratinocytes, while CD34, CD71, C-kit and involucrin were weakly expressed. C-kit is reported to be highly expressed in bone marrow stem cells, but weakly expressed in mouse skin keratinocyte stem cells [21,32]. CD34 was proven to be a marker for hair follicle bulge keratinocyte stem cells in mouse [6,8,14], and a recent report indicated that it was weakly expressed in human hair follicle keratinocyte stem cells [41]. Our data for C-kit and CD34 were compatible with previous reports. These results indicated that ABCG2-positive keratinocytes express several potential stem cells markers; this cell population shows a phenotype of basal layer keratinocytes, and they are not differentiated keratinocytes.

Clonal analysis was used to evaluate keratinocyte clones formed *in vitro*. According to the percentage of aborted colonies form by the progeny of the seeding cell [37], keratinocyte clones produced in tissue culture can be categorized as holoclones, meroclones and paraclones. Holoclones showed extensive proliferative potential in *in vitro* culture, with a mean population doubling of 120 to 160 in human skin keratinocytes [37]. Recently, a transplantation assay of holoclones in the rodent model [42] indicated that holoclones maintained their stemness and multipotentiality in tissue culture. Therefore, holoclones can be considered as containing keratinocyte stem cells [2,9,15,42]. Our data show that in the ABCG2-positive keratinocyte population approximately 74% of cells were holoclones, while there was less than 5% of holoclones in ABCG2-negative cells. We feel confident that ABCG2-positive keratinocytes exhibit a major subset of keratinocyte stem cells in human interfollicular skin. Our results also showed that the ABCG2-positive cells had population doublings of 120 to 140, which is similar to the previous proliferative potential report of holoclones [37].

With the *in vitro* epidermal reconstitution assay, we demonstrate that the skin generated by ABCG2-positive keratinocytes exhibited significantly thicker pluristratified epidermis, and a more well ordered epidermal structure containing the stratum granulosum and stratum corneum, than the skin generated by ABCG2-negative keratinocytes. This finding indicates that ABCG2-positive keratinocytes are not only capable of self-renewal but of giving rise to hierarchical differentiating progeny, which are traits of keratinocyte stem cells. The observed high proliferative potential of ABCG2-positive keratinocytes, as indicated by CFE assay and clonal analysis, suggests that these cells may contribute to this enhanced skin thickness and well ordered epidermis structure.

In previous studies, the ABCG2 transporter has been suggested to be the molecular determinant of the SP cell phenotype in many tissues [20,22,23]. However some studies showed that ABCG2 might not characterize SP keratinocytes [20,28,31,34], which were different from the reports in other tissues and organs. The complex and sensitive procedure of the Hoechst 33342 exclusion assay may contribute to this controversy [28]. Also, Hoechst 33342 is a DNA intercalating agent that results in significant cellular toxicity, and may have caused RNA degradation in the sorted cell population [39,43]. A recent study also suggests that Hoechst 33342 could affect cell differentiation [43], which could further complicate the interpretation of SP keratinocyte studies. In this study, we have concentrated on the expression of ABCG2 in human interfollicular skin. We used a monoclonal antibody to ABCG2 (clone 5D3) to sort out ABCG2-positive keratinocytes, which provides more stable and consistent data than the Hoechst dye exclusion assay.

The robust identification of keratinocyte stem/progenitor cells has major practical importance for generating a better characterization of the biochemical properties, and growth and proliferation characteristics of human interfollicular stem cells that sustain human epidermis for many decades. The efficient selection of clonogenic keratinocytes also has many valuable clinical applications, such as in severe burns, corneal defect [44] and stem cell therapy and gene therapy in recessive dystrophic epidermolysis bullosa patients [45]. Keratinocyte stem cells have been identified *in vivo* by label retention assays or *in vitro* by clonogenic assays [1,2,9]. However, both of these methods are too destructive to be useful for isolation and purification of viable keratinocyte stem cells. On the other hand, many molecular markers have been proposed to enrich for keratinocyte stem cells, including p63, keratin 19, α6-integrin, β1-integrin, CD71, desmoplakin, Rac1, MTS24 and survivin, or a combination of cell kinetics and surface markers [3,12,13,15-18,46,47]. However, no single marker or method has been universally adopted for identifying and isolating keratinocyte stem cells, making the search for additional molecular markers worth pursuing [19,46].

Cultured epithelial grafts have been developed by culturing keratinocytes in a Petri dish, on fibrin matrices or on human/pig plasma [48-50]. The advantage of using a fibrin matrix is that it maintains the stemness of keratinocyte stem cells, which is most important for skin transplantation and regeneration. In this study, pig plasma was used to support keratinocyte growth and served as a dermal scaffold for human fibroblasts. The high long-term take rate of cultured human epidermal grafts in nude mice suggests that this protocol allows functional analysis of epidermal stem cells *in vivo*.

In this study, the human grafts derived from ABCG2-positive, a6-integrin-positive and control keratinocytes showed excellent take and survived up to 20 weeks. After 12 weeks, the epidermis derived from ABCG2-positive keratinocytes showed thicker epidermis cell layers than in the control and α6-integrin-positive groups. This difference might be related to the different proportions of clonogenic cells present in the deriving keratinocytes, as our previous results suggested high proportions of clonogenic cells within the ABCG2-positive population.

## Conclusions

We have demonstrated that ABCG2-positive keratinocytes can be isolated from human skin interfollicular epidermis, and that these cells have an undifferentiated keratinocyte phenotype and retain a range of biomarkers that suggest they can be interpreted as the interfollicular (non-hair-follicle) stem cells of the epidermis. Since the long-term durability of skin keratinocyte transplantation depends on the reserve and maintenance of keratinocyte stem cells in the skin grafts [50], the identification of ABCG2-positive keratinocytes is expected to prove useful for clinical applications, such as in wound healing and generating better quality skin replacement.

## Abbreviations

ABC: ATP binding cassette
CFE: colony-forming efficiency
DMEM: Dulbecco’s modified Eagle’s medium
FACS: fluorescence-activated cell sorting
FBS: fetal bovine serum
PBS: phosphate-buffered saline
SP: side population
